# A Case of Febrile Polyphagia With an Underlying Paraneoplastic Limbic Encephalitis

**DOI:** 10.7759/cureus.42947

**Published:** 2023-08-04

**Authors:** Rita João Soares, Mafalda Miranda, Nuno Monteiro, Giovana Ennis, Joana Silva Marques

**Affiliations:** 1 Department of Internal Medicine, Centro Hospitalar Tondela-Viseu, Viseu, PRT; 2 Department of Internal Medicine, Casa de Saúde São Mateus, Viseu, PRT

**Keywords:** onconeural antigens, neuropsychiatric symptoms, carcinosarcoma of the uterus, paraneoplastic neurological syndrome, limbic encephalitis

## Abstract

A 57-year-old female with a history of malignant mixed Müllerian tumors of the uterus and ovaries developed a fever of unknown origin and neuropsychiatric symptoms. Her EEG showed slow activity in the left temporal region, and brain magnetic resonance imaging revealed limbic encephalitis, leading to the diagnosis of classic paraneoplastic limbic encephalitis (PLE). During our investigation into the underlying cause of the patient's condition, we conducted a PET-CT scan, which revealed the presence of several hypermetabolic lymph nodes. One of these lymph nodes underwent a biopsy, and the results confirmed the presence of metastatic cells, indicating the likelihood of carcinoma, most probably adenocarcinoma of the gynecological tract. PLE should be considered as one of the differential diagnoses in patients with a history of cancer and acute-to-subacute neuronal and psychiatric dysfunction.

## Introduction

Paraneoplastic limbic encephalitis (PLE) is a disorder characterized by a combination of neuropsychiatric symptoms resulting from the indirect impact of a tumor situated outside the central nervous system (CNS) [1-3]. The diagnosis should be considered in any patient with a gradual onset of mood changes, psychosis, cognitive decline, memory impairment, sleep disturbances, irritability, or seizure activity (typically complex-partial seizures) [1,4]. PLE usually involves the mesial temporal lobe and limbic mesial cortical structures, including the cingulate gyrus, orbitofrontal cortex, and mammillary bodies [4,5].

PLE is believed to be caused by an autoimmune mechanism. Antigens present in the CNS may bear a resemblance to antigens found ectopically on tumors, known as onconeural antigens. When these onconeural antigens are recognized beyond the nervous system, it triggers an immune response intended to protect the body but unintentionally causes harm to CNS tissue when antibodies and cytotoxic T-cells breach the blood-brain barrier and interact with neural cells [1,2]. In approximately 90% of patients with PLE, autoantibodies that target onconeural antigens found outside the CNS are present [4,5]. The most frequently implicated tumor types are small-cell lung cancer, followed by testicular germ cell tumors and Hodgkin lymphoma [4,6]. 

## Case presentation

A 57-year-old female pharmacist presented to the emergency department after experiencing a fall from a height resulting in head trauma. Her medical history included migraines and osteoporosis. Additionally, she underwent a total hysterectomy with bilateral adnexectomy and bilateral pelvic and para-aortic lymphadenectomy five years prior due to a malignant mixed Müllerian tumor (MMMT) of the uterus and ovaries. Her medication regimen consisted of trimetazidine, glucosamine+chondroitin, ginkgo biloba, and a vitamin supplement. Her neurological examination showed no focal signs, there were no changes in oculomotor function or cerebellar coordination, and there were no indications of cortical dysfunction, such as aphasia, sensory neglect, visual field defects, or temporal or spatial disorientation. However, the patient displayed disorganized and incoherent speech with persecutory delusions, along with amnesia for recent events. There were no signs of motor lateralization, and no language deficits were observed, but there were occasional sudden involuntary movements (myoclonic-like). According to the patient's family, these symptoms have been progressively worsening over the course of a couple of months. Similar episodes had occurred in the previous year but spontaneously resolved. Upon examination in the emergency department, the patient’s vital signs were within normal ranges. She reported pain at the site of head trauma and in her left lower limb, although there were no external signs of trauma. A second-degree burn on her right forearm, which had been present for two weeks and was healing without signs of inflammation or infection, was observed. Further diagnostic tests, including blood analysis, showed an increase in liver enzyme levels, approximately ten times the upper limit (Table 1). LDH levels were also found to be elevated. Abdominal CT ruled out acute abdominal pathology, and serological tests for HIV, HCV, and HBV were negative. Cranial CT, chest X-ray, pelvic X-ray, and upper abdominal ultrasound were conducted, with no significant abnormalities detected. The patient received evaluation from a psychiatry specialist, who determined that there were no psychotic symptoms but rather a presentation consistent with post-traumatic brain injury and anterograde amnesia. Due to the suspicion of encephalopathy during the neurology observation, a lumbar puncture was performed to investigate potential neuro-infectious, inflammatory, and neoplastic causes of these neurological symptoms. The procedure was uneventful and showed a slight elevation in protein concentration as well as a slight increase in leukocytes, as shown in Table 1. However, microbiological cultures of cerebrospinal fluid (CSF) were negative, and an extensive search for DNA of specific bacteria, viruses, and fungi also yielded negative results. An electroencephalogram (EEG) was conducted in the emergency department to aid in the evaluation of this encephalopathy. It revealed a normal tracing; this result indicated that there were no abnormal electrical patterns or epileptiform activities detected in the brain at the time of the test. The patient was admitted to an internal medicine ward for further evaluation and monitoring.

**Table 1:** Laboratory test ALT: Aspartate aminotransferase; AST: Aspartate transaminase; LDH: Lactate dehydrogenase; CRP: C-reactive protein; ESR: Erythrocyte sedimentation rate; TSH: Thyroid stimulating hormone; CSF: Cerebrospinal fluid

	Values	Normal range
Leucocytes	6.30×10^9^/L	4.50 to 11.50
Neutrophils	4.8×10^9^/L	
Platelet count	156x10^9^/L	150 to 450
Hemoglobin	11.5 g/dL	12.0 to 15.0
Urea	37 mg/dL	14 to 42
Creatinine	0.8 mg/dL	0.5 to 1.2
Sodium	141 mEq/L	136 to 145
Potassium	4.8 mEq/L	3.4 to 4.4
Glucose	95 mg/dL	74 to 106
AST	336 UI/L	3 to 31
ALT	453 UI/L	3 to 31
LDH	1090 UI/L	200 to 480
CRP	3.94 mg/dL	< 0.50
TSH	0.073 mUI/L	0.350 to 5.500
Free T4	1.1 ng/dL	0.9 to 1.8
HIV, HCV, HBV antibodies and HBsAg	Not detected	
CSF glucose	54 mg/dL	50 to 80
CSF protein	68 mg/dL	15.0 to 40.0
CSF leucocytes	15 leuc/mm^3 ^(with 85% predominance of mononuclear cells, and 15% neutrophils)	

**Figure 1:**
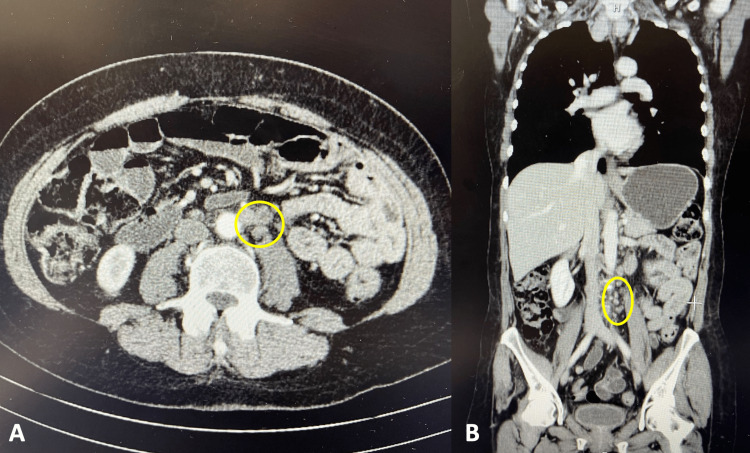
Patient's abdominal CT (A: axial plane; B: coronal plane) Abdominal CT shows several small lymph nodes in the intercavoaortic and lateroaortic regions (marked).

**Figure 2:**
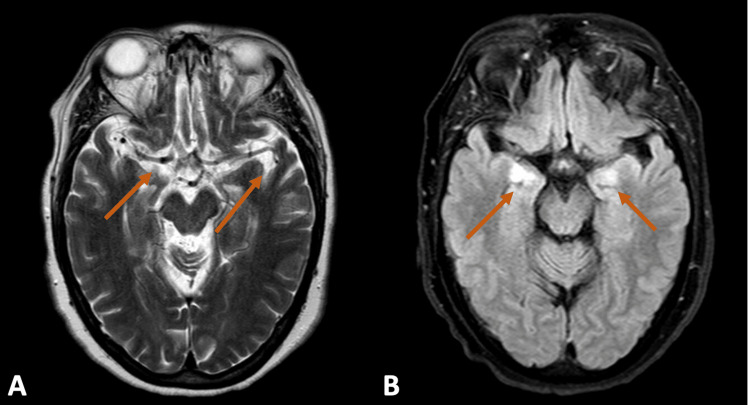
Patient's brain MRI A: Axial T2 shows a bilateral hyperintensity of the temporal lobes. B: Axial T2 FLAIR showing hyperintensity in the medial temporal lobe bilaterally.

**Figure 3:**
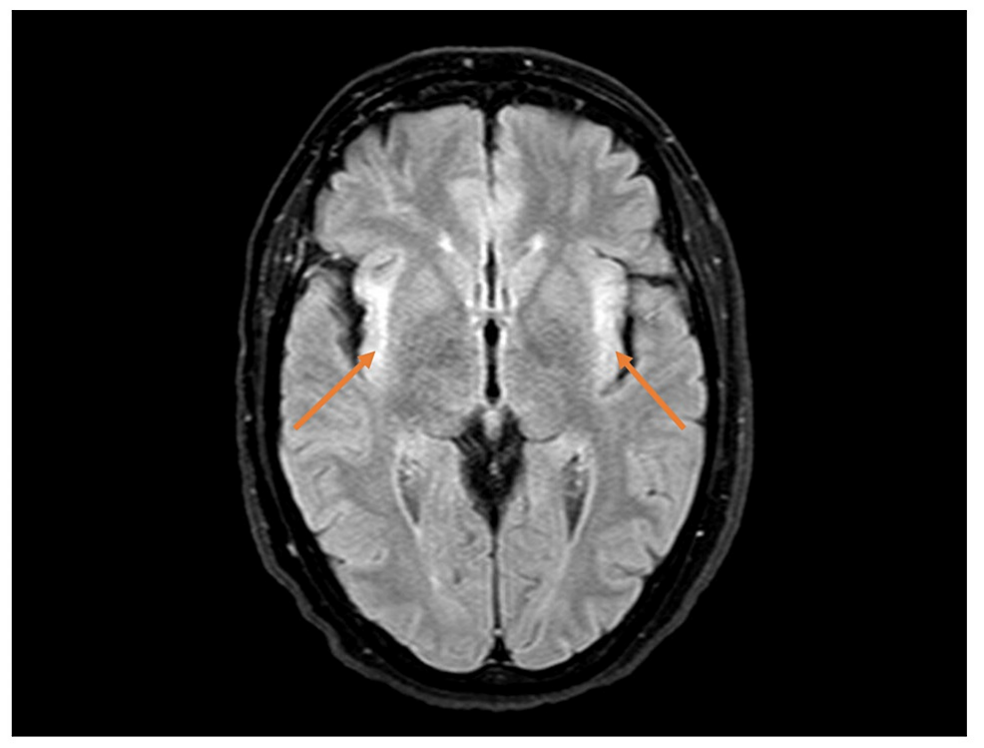
Patient's brain MRI Axial T2 flair showing bilateral hyperintensity of the insular cortices

As she developed an unexplained persistent fever during the early days of admission, a thorough diagnostic workup was initiated to identify the underlying cause. Blood cultures were performed, and the results showed the presence of *Staphylococcus epidermidis*. This finding suggested a possible connection to her burn wound on her arm. She completed a 10-day course of penicillin, but the fever persisted without responding to antipyretics. Additionally, she developed symptoms of compulsive polyphagia (excessive eating), frontal disinhibition with impulsive behavior, such as stealing and hoarding sugar packets from other patients, and the ingestion of non-prescribed medication in excessive amounts, facilitated by a friend who visited her at the hospital. The combination of irritability and mood swings added to the complexity of her presentation, and given these abnormal neurological findings and the changes in her behavior, further investigation and evaluation were needed. Subclinical hyperthyroidism was detected in the laboratory tests, and an ultrasound of the thyroid revealed an enlarged, multinodular thyroid gland. A biopsy of a nodule was performed, which showed a benign follicular nodule. Thiamazole was prescribed for her thyroid condition. Blood and urine cultures were repeated and yielded negative results. Colonoscopy did not reveal any abnormal findings, while endoscopy identified a white plaque lesion in her upper esophagus, which was biopsied and found to be positive for vascular congestion but without dysplasia. Serology and ELISA tests for brucella and blood serology for *Coxiella burnetii* were negative. Wilson's disease was considered a potential diagnosis due to abnormalities in serum aminotransferases and neuropsychiatric symptoms, but both serum and urinary copper levels were within normal ranges. Acute endocarditis was ruled out based on transthoracic and transesophageal echocardiograms. A CT scan of her chest, abdomen, and pelvis revealed several small lymph nodes in the intercavoaortic and lateroaortic regions, with the largest measuring 16 mm, but no other abnormal findings were observed (Figure 1). Due to her history of a Müllerian tumor, a breast ultrasound was performed and showed no abnormalities, while a mammogram was recommended but not performed due to the patient's lack of cooperation. She also underwent a bone marrow aspiration and biopsy (medulogram and medullary biopsy), which revealed normal cellularity and morphology. Considering her behavioral disinhibition, a brain CT scan was conducted and found to be normal. Subsequently, a brain magnetic resonance imaging (MRI) study showed hyperintense signals on T2-weighted and T2-FLAIR images in the temporal (Figure 2) and insular regions (Figure 3), particularly the subinsular tonsils, without restricted diffusion or mass effect. Paraneoplastic and herpetic encephalitis were suggested as possible causes, although herpetic encephalitis was less likely due to the absence of restricted diffusion in the affected regions [1,3]. Non-specific *gliotic foci* were also identified in the right anterior frontal subcortical white matter and the right cerebellar hemisphere. Another EEG was performed, showing preserved baseline rhythm with slow activity in the left temporal region for a restricted duration, suggesting a functional deficiency in this region, and a second lumbar puncture was carried out to collect neuronal antibodies such as antineuronal nuclear antibody type 1 (ANNA-1) (anti-Hu), amphiphysin, collapsin response-mediator protein-5 (CRMP-5)-IgG, ANNA-2, and anti-Ma, which also turned out to be negative. Based on the suggestion of the Neurology department, immunoglobulin therapy and corticosteroids were initiated, resulting in a slight improvement in the patient's clinical condition. A follow-up MRI scan one month later showed less prominent hyperintensities in the insular/subinsular and temporomesial regions, which appeared atrophic. A PET-CT scan was requested and revealed multiple hypermetabolic lymph nodes. A fine-needle aspiration (FNA) biopsy of the left cervical lymph node confirmed metastasis by a carcinoma, likely adenocarcinoma, although the immunophenotyping of the lymph node was within normal limits. The immunohistochemical profile using cytokeratins suggested the breast, lung, or gynecological tract as the primary sites. The patient's case was discussed in a multidisciplinary meeting, and it was determined that due to the rapid progression of the disease and the deterioration of the patient's condition, no specific treatment for the neoplasm was recommended. Her neuropsychiatric symptoms worsened, leading to increased dependence on caregivers, and she eventually succumbed to nosocomial pneumonia one year later.

## Discussion

To the best of our knowledge, we present the first case of paraneoplastic limbic encephalitis resulting from metastatic MMMT of the uterus and ovaries, based on a manual search conducted on PubMed and Google using the terms "Malignant mixed Müllerian tumor" and "Paraneoplastic Limbic Encephalitis."

Among patients presenting with a clinical picture of limbic encephalitis, viral encephalitis, paraneoplastic encephalitis, and encephalitis associated with antibodies to cell surface antigens such as voltage-gated potassium channels (VGKC) antibodies (which can be paraneoplastic or not) are the three disorders initially considered more frequently [3,7]. PLE is a rare disorder characterized by personality changes, irritability, depression, seizures, memory loss, and, in some cases, dementia. The diagnosis of PLE presents significant challenges owing to the absence of consistent clinical markers. Often, the symptoms precede the diagnosis of cancer or can mimic other cancer-related complications, such as brain metastases, toxic and metabolic encephalopathies, infections (especially herpes simplex encephalitis), and the side effects of cancer therapy. The progression of symptoms in PLE can vary, with some cases showing rapid progression over days or weeks while others may have a more indolent presentation over months [4,5,8,9]. In line with the literature, the patient we are describing developed a neuropsychiatric condition characterized by polyphagia, memory loss, and mood swings associated with a fever of unknown origin. She had a history of total hysterectomy with bilateral adnexectomy and bilateral pelvic and para-aortic lymphadenectomy five years ago due to malignant mixed Müllerian tumors in the uterus. MMMTs of the female genital tract are uncommon yet highly aggressive tumors characterized by biphasic histology. They primarily originate in the uterus, with less frequent occurrences in the ovaries, fallopian tubes, and vagina. The overall five-year survival rate is only 30 ± 9% for all stages, and there is an exceptionally high recurrence rate (50%-80%) following surgery [10].

A comprehensive diagnostic evaluation usually involves electroencephalography, cranial magnetic resonance imaging, and a cerebrospinal fluid examination. However, it's important to note that the abnormalities detected in these tests may be nonspecific [1]. EEG findings often show focal or generalized slowing and/or epileptiform activity, with the most pronounced involvement seen in the temporal regions [4,8]. CSF analysis also frequently reveals nonspecific abnormalities but is crucial for ruling out infectious and carcinomatous processes [4]. It assists in the diagnosis of PLE in two ways. First, a negative cytological analysis for malignant cells, combined with the absence of meningeal enhancement on the MRI, helps to exclude leptomeningeal metastases. Second, the detection of inflammatory abnormalities such as pleocytosis, intrathecal synthesis of IgG, and oligoclonal bands supports the diagnosis of an inflammatory or immune-mediated neurological disorder [8]. MRI can reveal areas of T2/fluid-attenuated inversion recovery (FLAIR) hyperintensity in the medial temporal lobe. It is uncommon to observe contrast enhancement in these regions [4,8,11]. The role of PET/CT in detecting malignancy in PLE is still being explored, and there have not been any well-conducted clinical trials specifically addressing this aspect. However, fused PET and CT images have the potential to provide valuable information within a single study, including the presence and precise location of a viable tumor [12]. The prevalence of antineuronal antibodies in patients with PLE has not been extensively studied. The detection of one or more paraneoplastic autoantibodies can aid in the early diagnosis of PLE and guide the investigation of an underlying malignancy. However, it's important to note that in cases where the symptoms suggest limbic dysfunction and neurodiagnostic findings are typical, the absence of detectable paraneoplastic antibodies does not exclude the diagnosis of PLE, nor does it eliminate the necessity of conducting a thorough search for hidden malignancies [4].

Our patient’s MRI demonstrated signs consistent with limbic encephalitis, while the EEG and CSF analyses were nonspecific. It was only through the PET/CT scan, which revealed hypermetabolic lymph nodes, that we could ultimately confirm the presence of an underlying tumor and reach a diagnosis. The treatment of PNS typically involves the use of corticosteroids, immunosuppressive drugs, and plasma exchange therapy. However, their effectiveness is often reported to be low. The most effective approach to treating PNS is to address the primary malignancy through antitumor therapy. However, neuronal damage is extensive and irreversible, and in patients with prolonged neurological symptoms, achieving symptom improvement is usually challenging [4,6,13]. Early identification and targeted treatment of the underlying neoplasm, along with immunotherapy, are crucial for neurological recovery [8]. Unfortunately, our patient had a poor prognosis due to prolonged and fixed neurological symptoms. The patient's eligibility for antitumor treatment was discussed in a collaborative meeting with oncologists, neurologists, and gynecologists. Considering the severity of the neurological decline and the limited potential for beneficial outcomes from antitumor treatment, it was decided that the patient would not be eligible for such therapies.

## Conclusions

The literature on paraneoplastic limbic encephalitis is limited primarily because of its rare occurrence.

The purpose of this case report is to raise awareness about the importance of considering paraneoplastic limbic encephalitis as a potential diagnosis in patients with a history of cancer who present with acute-to-subacute neuronal and psychiatric dysfunction.
